# PCR-based detection and serovar identification of *Salmonella* in retail meat collected from wet markets in Metro Manila, Philippines

**DOI:** 10.1371/journal.pone.0239457

**Published:** 2020-09-30

**Authors:** Pauline Dianne M. Santos, Kenneth W. Widmer, Windell L. Rivera

**Affiliations:** 1 Pathogen-Host-Environment Interactions Research Laboratory, Institute of Biology, College of Science, University of the Philippines Diliman, Quezon City, Philippines; 2 International Environmental Research Institute, Gwangju Institute of Science and Technology, Gwangju, Republic of Korea; University of Bologna, ITALY

## Abstract

This study aimed to detect *Salmonella* from retail meat collected from nine wet markets in Metro Manila, and identify the subtypes of *Salmonella* isolates using molecular serotyping assays from previously developed primers. Of the 720 collected meat samples, 57.64% were found to be *Salmonella-*contaminated. The most predominant serogroup was *Salmonella* O:3, and *Salmonella* serogroups O:4, O:6,7, O:8, O:9, and undetermined serogroups were also found. Most frequently detected isolates in bovine meat were *S*. 3:e,h:1,6 (putative identity: *S*. Anatum) and *S*: 4:e,h:1,2 (putative identity: *S*. Saintpaul), in porcine meat was *S*. 3:e,h:1,6 (putative identity: *S*. Anatum), and *S*. 8:i:z6 (putative identity: *S*. Kentucky) was common in poultry products. This study also demonstrated retail meat samples were contaminated with multiple *Salmonella* serogroups and serovars. This is the first Philippine study that utilized PCR-based assays to characterize *Salmonella* isolates down to a serovar level and provides baseline information regarding *Salmonella* prevalence and serovar distribution in retail meat. Molecular serotyping performed in this study can be used as an alternative approach to traditional serotyping in surveillance of *Salmonella* in the Philippines since the latter is expensive, time-consuming, and requires skilled technicians.

## Introduction

*Salmonella* is considered one of the most significant pathogens associated with food-borne diseases and outbreaks in the world [1,2]. It is estimated to cause approximately 93.8 million human infections and 155,000 deaths annually worldwide [3] and major economic losses in poultry and livestock industries [4]. The genus *Salmonella* is classified into serovars, which are based on the presence of somatic (O) and flagellar (H) antigens. Serotyping has been essential in public health monitoring of *Salmonella* infection for more than 80 years [5]. White-Kauffman-Le Minor (WKL) scheme contains the organized list of 2,659 serovars, which were characterized by serological agglutination of 46 somatic and 119 flagellar antigens [6,7]. However, serological agglutination reaction is limited by antisera production, time-consuming, extremely complex procedure, high cost, and at times inconsistent results due to the presence of capsule phenotype, rough phenotype, or non-expression of flagellar phase [8]. These limitations led to the development of molecular-based serotyping using multiplex PCR, which allows for the simultaneous, rapid, sensitive, and specific detection of various *Salmonella* genes that express somatic [9] and flagellar antigens [10,11]. Several of these primer pairs were tested for specificity and sensitivity by the National Institute for Public Health and the Environment reference laboratory in The Netherlands (RIVM) as an alternative approach to gold standard [12].

In the Philippines, surveillance data regarding *Salmonella* serogroup and serotype distribution are limited. *Salmonella* serotyping is not routinely performed due to previously mentioned disadvantages. Most *Salmonella* isolates from clinical and environmental settings collected in different parts of the country are submitted to the Antimicrobial Resistance Surveillance Reference Laboratory (ARSRL) in the Research Institute for Tropical Medicine (RITM) for serotyping. Molecular subtyping of *Salmonella* was previously performed in studies by Soguilon & Rivera, Ng & Rivera, and Calayag et al. [13–15] in slaughtered swine and meat samples collected from Metro Manila, Philippines. However, these studies were limited to serogrouping and detection of *Sdf-I* expressing gene. Hence, a study that determines *Salmonella* serotype using molecular subtyping is needed.

This study aimed to detect *Salmonella* spp. from various raw meat and meat products collected from nine selected Metro Manila wet markets using PCR-based detection assays described by Ng & Rivera [14] and Soguilon & Rivera [13], and classify *Salmonella* based on their O-serogroups and serotypes using PCR-based serotyping assays. This study can contribute to the epidemiology of salmonellosis in the Philippines, particularly on reservoirs of clinically important serotypes. Data regarding the *Salmonella* prevalence and serotype distribution in retail meat are significant in the identification of important *Salmonella* reservoirs and initiation of long-term prevention and control measures.

## Materials and methods

### Sample collection

Two hundred seventy-four (274) raw meat (minced beef = 90, chicken wings = 94, ground pork = 90) and 446 processed meat (bacon = 90, burger patties = 70, Filipino-style luncheon meat = 90, sliced ham = 85, Filipino-style sausage = 96, salami = 15) samples were collected from nine wet markets in Metro Manila from 2015–2016. A total of 80 samples were purchased randomly at different stalls in each wet market with varying frequencies depending on market availability of meat products. Raw meat samples were defined as animal muscle and fat that were chopped, minced, or ground, while processed meat samples were defined as meat samples which underwent salting, curing, addition of seasonings and other food materials, and/or heat treatment. Samples were stored in a cooler at approximately 5°C during transport and immediately processed in the laboratory for the pre-enrichment procedure. All raw meat products were freshly slaughtered from a local city abattoir, while processed meat products were either prepared from wet markets or local meat processing plants. Each market location was given a precursory survey of the conditions, operation facilities, and general practices of the workers at the time of collection. Quality of the market sanitation and practices were ranked on an ordinal scale of five values ranging from poor to very good (S1 Appendix).

### *Salmonella* detection and isolation

The culture detection technique for *Salmonella* detection was based on the modified ISO 6579:2002 protocol previously described by Soguilon-del Rosario & Rivera and Ng & Rivera [13–14]. Briefly, 25 g meat sample was aseptically manually homogenized and inoculated into 225 ml buffered peptone water (BPW; BD Diagnostics System Difco^TM^ USA) in a sterile bottle. The pre-enrichment culture was agitated for at least 2 min and incubated at 37°C for 18–24 h. One hundred microliters of pre-enriched culture was inoculated to 10 ml Rappaport Vassiliadis (RV; BD Diagnostics System Difco^TM^ USA) broth in duplicate and incubated at 42°C for 18–24 h. A loopful of culture medium from the incubated RV broth was streaked onto Brilliant Green (BG; BD Diagnostics System Difco^TM^ USA) agar and Xylose Lysine Deoxycholate (XLD; BD Diagnostics System Difco^TM^ USA) agar plates and incubated at 37°C for 18–24 h. Typical *Salmonella* colonies were selected and isolated from both BG agar and XLD agar. Each isolate was streaked onto NA (nutrient agar; BD Diagnostics System Difco^TM^ USA) slants and incubated at 37°C for 24 h. A single colony from the NA slant was inoculated to 1 ml Tryptic Soy Broth (TSB; BD Diagnostics System Bacto^TM^ USA). After incubation at 37°C for 24 h, 330 μl of 100% glycerol was added per isolate, vortexed, and stored at -20°C until use. RV broths and putative *Salmonella* isolates grown on NA slants were saved for subsequent DNA extraction.

### Revival and purification

From *Salmonella* glycerol stocks, 50 μl stock culture was inoculated to 1 ml TSB and incubated at 37°C for 24 h. A loopful of cultured *Salmonella* in TSB was streaked onto XLD agar plate. After incubation, a single typical *Salmonella* colony isolate was selected for inoculation into two microcentrifuge tubes with 1 ml TSB each. Isolation steps using XLD agar plates were repeated until a single colony of presumptive *Salmonella* was obtained. One of the TSB tubes was subjected to DNA extraction, while the other was stored as a glycerol stock.

### DNA extraction

Three different template extraction protocols (TEP) were performed in this study, and DNA templates were extracted in the following *Salmonella* culture steps: (TEP I) after 18–24 h incubation of pre-enriched samples in RV broth, (TEP II) after 18–24 h incubation of putative *Salmonella* isolates inoculated in NA slants, and (TEP III) after revival and purification of *Salmonella* isolates from glycerol stocks. TEP I was intended for the rapid identification of *Salmonella* within samples. Data regarding *Salmonella* prevalence were gathered from TEP I and TEP II, while results of TEP III verified that the revived and purified isolates were *Salmonella* and was used as templates to characterize isolate serotypes.

Two milliliters of culture from RV broth (TEP I) and 1 ml of *Salmonella* culture in TSB (TEP III) were centrifuged at 15,330 ×g for 5 min [13]. The resulting supernatant was discarded while the pooled bacterial pellet was suspended in 1 ml phosphate-buffered saline (PBS). The bacterial suspension was vortexed for 1 min and centrifuged at 15,330 ×g for 5 min. The resulting pellet was suspended in 50 μl and 100 μl sterile Tris EDTA (TE) buffer for RV broth and TSB, respectively. For TEP II, a loopful of *Salmonella* was collected from the nutrient agar slant and transferred to a microcentrifuge tube containing 100 μl sterile Tris EDTA (TE). Bacterial suspensions were boiled at 100°C for 10 min using a dry heating block. Resulting lysed cells were centrifuged at 2,656 ×g for 5 min. The supernatants were transferred to a new microcentrifuge tube and stored at -20°C until use.

### Molecular detection and characterization

Four-step PCR-based assays using T100 Thermal Cyclers (Bio-Rad, USA) were employed in molecular detection and serotyping of *Salmonella* from meat samples. Step 1 involved the detection of *Salmonella* spp. using PCR that targeted the *invA* gene in DNA extracted from TEP I and II. In step 2, *Salmonella* positive samples were run as multiplex PCR experiments using DNA extracted from TEP I and TEP III. Step 3 determined the presence of phase 1 flagellar antigen (H1) encoding genes while step 4 determined the presence of phase 2 flagellar (H2) antigen and *Sdf-I* encoding genes. The results of the last three PCR assays defined the banding pattern of each *Salmonella* isolate, which would correspond to a serovar identity. Steps 3 and 4 analyzed DNA extracted from TEP III.

#### Polymerase Chain Reaction (PCR)-based detection of *Salmonella* spp.

The amplification of the *invA* gene was performed in a 12.5 μl reaction volume. Each reaction consisted of 6.25 μl 2× Promega Green master mix (1 U *i*-*Taq*^TM^ DNA polymerase, 2× PCR buffer, 3 mM MgCl_2_ and 0.4 mM dNTPs; Promega, USA), 0.5 μl each of forward and reverse primer (10 uM; Table 1, Reaction group 1), 1 μl DNA template and 4.25 μl of PCR water. PCR was performed using the following conditions: initial denaturation at 95°C for 3 min; denaturation at 95°C for 30 s, annealing at 60°C for 30 sec, extension at 72°C for 30 sec, repeated for 35 times, and a final extension at 72°C for 5 min.

**Table 1:** Primers for *Salmonella* identification and characterization. List of primers for *Salmonella* detection, serogrouping, and serotyping assays.

Primer name	F/ R	Sequence (5’-3’)	Reaction group	Target	Amplicon length (bp)	Reference
*invA-1*	F	ACAGTGCTCGTTTACGACCTGAAT	1	*Salmonella* spp.	244	[16]
*invA-2*	R	AGACGACTGGTACTGATCGATAAT	1			
*abe1*	F	GGCTTCCGGCTTTATTGG	2	O:4 (B)	561	[9]
	R	TCTCTTATCTGTTCGCCTGTTG	2			
*wbaD-*	F	ATTTGCCCAGTTCGGTTTG	2	O:6,7 (C_1_)	341	
*manC*	R	CCATAACCGACTTCCATTTCC	2			
*abe2*	F	CGTCCTATAACCGAGCCAAC	2	O:8 (C_2_-C_3_)	397	
	R	CTGCTTTATCCCTCTCACCG	2			
*wzx- wzyE1*	F	GATAGCAACGTTCGGAAATTC	2	O:3 (E_1_-E_4_)	281	
	R	CCCAATAGCAATAAACCAAGC	2			
*Tyv*	F	GAGGAAGGGAAATGAAGCTTTT	2	O:9 (D1)	614	[17]
	R	TAGCAAACTGTCTCCCACCATAC	2			
*prt*	F	CTTGCTATGGAAGACATAACGAACC	2	O:2; O:9	256	
	R	CGTCTCCATCAAAAGCTCCATAGA	2	(A; D)		
*P1*	F	TTATTAGGATCGCGCCAGGC	2	*oriC* gene	163	[18]
*P2*	R	AAAGAATAACCGTTGTTCAC	2			
*S60*	F	GCAGATCAACTCTCAGACCCTGGG	3		-	[11]
*as-z10*	R	CGTCGCAGCTTCTGCAACC	3	*fliC-z10*	448	
*as-r*	R	AAGTGACTTTTCCATCGGCTG	3	*fliC-r*	281	
*as-i*	R	ATAGCCATCTTTACCAGTTCC	3	*fliC-i*	253	
*as-e,h*	R	AACGAAAGCGTAGCAGACAAG	3	*fliC-e,h*	200	
*as-b*	R	CGCACCAGTCYWACCTAAGGCGG	3	*fliC-b*	169	
*G*	F	GTGATCTGAAATCCAGCTTCAAG	3	*G*	509	
	R	AAGTTTCGCACTCTCGTTTTTGG	3			
*D*	F	CCCGAAAGAAACTGCTGTAACCG	3	*fliC-d*	87	
	R	TGGATATCAGTATTGCTCTGGGC	3			
*F1mod*	F	CTTATGCCRATAATGGTACTACACTG	4	-	-	[10]
*R1mod*	R	TTTGACCAAYKYMGCGSCAT	4	*fljB-1,2*	388	
*R6*	R	CTCCTGTACTTCTGTTTTGGTTGTA	4	*fljB-1,6*	290	
*R7*	R	TAATCGCCATTTTTGTCGAG	4	*fljB-1,7*	190	
*Sense-Fe*	F	GGCAACCCGACAGTAACTGGCGATAC	4	-	-	
*as-Rx*	R	CCATCCTTAAAGGATACGGC	4	*fljB-e,n,x*	56	
*as-Rz15*	R	ATCAACGGTAACTTCATATTTG	4	*fljB-e,n,z15*	135	
*Sense-lw*	F	GTGGGGCAACMCTCAATACTG	4	*fljB-l,w*	241	
*as-lw*	R	CCTGCCACTTTCGTGGTTGC	4			
*Z6-F*	F	GCTGTGACAGTAGCTGCCAAT	5	*fljB-z6*	240	[8]
*Z6-R*	R	CGTACCAGCGGTCATAGACAC	5			
*Sdf-I*	F	TGTGTTTTATCTGATGCAAGAGG	6	*Sdf-I*	333	[19]
	R	CGTTCTTCTGGTACTTACGATGAC	6			

F-forward, R-reverse, g—g-complex. s-sense. as-anti-sense.

#### O-serogrouping of *Salmonella* spp

The O-serogrouping assay was performed in a 12.5 μl reaction. Each reaction was composed of 6.25 μl 2× KAPA2G Fast multiplex mix (1 U *i*-*Taq*^TM^ DNA polymerase, 1.5× PCR buffer, 3 mM MgCl_2_, and 0.2 mM dNTPs; Kapa Biosystems, USA), 0.25 μl each of 7 forward and reverse primer (10 uM; Table 1, Reaction group 2), 1 μl DNA template and 1.75 μl of PCR water. Multiplex PCR was accomplished using the following conditions: initial denaturation at 95°C for 3 min; denaturation at 95°C for 30 sec, annealing at 58°C for 30 sec and extension at 72°C for 1 min run in 35 cycles, and a final extension at 72°C for 5 min.

#### H-typing I assay

H-typing I assay was performed in a 12.5 μl reaction, wherein each reaction was composed of 6.25 μl 2× KAPA2G Fast multiplex mix (Kapa Biosystems, USA), 3 forward and 7 reverse primers (10 uM; Table 1, Reaction group 3), 1 μl DNA template and 2 μl of PCR water. Multiplex PCR was run using the following conditions: initial denaturation at 95°C for 3 min; denaturation at 95°C for 40 sec, annealing at 58°C for 30 sec and extension at 72°C for 30 sec for 30 cycles, and a final extension at 72°C for 7 min.

#### H-typing II assay

Samples were further characterized in H-typing II assays and primer pairs shown in Table 1 were used for these experiments. H-typing II assay was performed in a 12.5 μl reaction, which was composed of 6.25 μl 2× KAPA2G Fast multiplex mix (Kapa Biosystems, USA), 3 forward and 6 reverse primers (10 uM; Table 1, Reaction group 4), 1 μl DNA template and 0.75 μl of PCR water. Multiplex PCR was run using the following conditions: initial denaturation at 95°C for 3 min; denaturation at 95°C for 40 sec, annealing at 58°C for 20 sec and extension at 72°C for 20 sec for 30 cycles, and a final extension at 72°C for 7 min.

Serogroup O:9 was further characterized by a primer pair that detects the *Sdf-I* gene (Table 1, Reaction group 5), while samples with a missing band that determined their phase 2-flagellin were further characterized with z6 primers (Table 1, Reaction group 6). PCR reaction mix and conditions for amplification were similar to step 1.

### Gel electrophoresis

Amplicons from steps 1 and 2 PCR assays were run in 2% agarose gels (Promega, USA) with 15% GelRed^TM^ nucleic acid gel stain (Biotium, USA) at 280 V for 30 min (Bio-Rad, USA) using 1× Tris-Acetic Acid-EDTA (TAE) as running buffer. Amplicons from steps 3 and 4 PCR assays were run in 3% agarose gels (Promega, USA) with 15% GelRed^TM^ nucleic acid gel stain (Biotium, USA) at 280 V for 50 min (Bio-Rad, USA). Amplicon sizes were estimated using 100-bp plus molecular weight marker (Kapa Biosystems, USA). Gels were viewed under UV using a gel documentation system (Vilber Lourmat, France).

### Traditional serotyping

Serological agglutination assays of 16 selected *Salmonella* isolates with pre-determined antigenic formulae derived from molecular serotyping were outsourced to Antimicrobial Resistance Surveillance Reference Laboratory (ARSRL) at the Research Institute for Tropical Medicine (Metro Manila, Philippines).

### Data analyses

Fischer’s Exact Test was used to evaluate differences between the number of *Salmonella-*contaminated samples in raw and processed meat, as well as comparisons of *Salmonella* detection frequencies for culture versus PCR methods and different TEPs. Statistical tests were performed using IBM SPSS Statistics Version 20 and R 4.0.

## Results and discussion

In this study, we investigated the *Salmonella* prevalence in raw and processed meat samples collected from nine wet markets in Metro Manila, Philippines from 2015–2016 using PCR-based assay. We also characterized the serogroups and serotypes of *Salmonella* isolates using molecular serotyping assays developed by previous published studies [8,10,11,19]. These aims were achieved by performing three template extraction protocols (TEPs). Using TEP I, *Salmonella* detection in meat samples was achieved within 2 days, while traditional culture techniques requires 3–4 days to obtain negative results and 5–7 days to obtain confirmed positive results [20]. Putative *Salmonella* colonies from selective growth media were confirmed by extracting the DNA of these colonies grown in NA slants (TEP II). Combined results of PCR targeting *Salmonella* using DNA extracted using TEP I and II determined the prevalence of the pathogen in collected meat samples. Multiple *Salmonella* serogroups were detected in DNA extracted using TEPs I and II, thus, further isolation and purification steps were applied to revived *Salmonella* isolates. *Salmonella* serogroup frequencies were compared between DNA extracted from TEP I and III. Molecular serogrouping and serotyping assays were performed to DNA of *Salmonella* isolates extracted using TEP III.

### Presence and distribution of *Salmonella* in retail meat

A wet market is a type of traditional food retailer in the Philippines that offers affordable grains, fresh produce such as meat, fish, fruits, and vegetables [21]. However, this type of market has been linked to major diseases due to poor hygienic conditions [22]. Various studies have detected wet markets as an important source of *Salmonella*, where prevalence ranged from 6.8–87.5% in various retail meat [13,22–30]. This study found that 415 (57.64%) of 720 purchased raw and processed meat products were contaminated with *Salmonella* using PCR-based *Salmonella* detection (Table 2). Extraction protocols TEP I (n = 392) and TEP II (n = 306) yielded different frequencies of *Salmonella* positive meat samples. Despite utilizing different DNA extraction protocols, there were no statistically significant differences in the detection of *Salmonella* between TEP I and TEP II (p = 0.162) in both processed and raw meat samples. Also, combining the number of *Salmonella* positive samples from TEP I and II (overall) had resulted in relatively higher *Salmonella* positive results (n = 415), but the overall *Salmonella* prevalence in retail meat was not significantly different when compared to the results of TEP I (p = 0.8314) and TEP II (0.1075). TEP I detected relatively higher *Salmonella* positives samples than TEP II, however, TEP I also missed 23 positive samples that were confirmed by TEP II. This suggests that utilizing both TEP I and TEP II was important to have a relatively higher sensitivity in the detection of *Salmonella*. Additionally, comparisons for culture method detection of *Salmonella* versus TEP I (p = 0.453) and TEP II (p = 0.519) in both raw and processed meat samples also had no significant differences. This *Salmonella* prevalence in this study was relatively higher compared to previous studies that detected *Salmonella* in the Philippines, such as Soguilon and Rivera (30.63%, n = 320) [13], Baldrias & Capistrano (19.76%, n = 167) [24], Vismanos et al. (8.96%, n = 212) [25], and Balala et al. (9.33%, n = 150) [31], and other Asian countries, such as Vietnam (35.52%, n = 608) [32], China (6.8%, n = 1414) [23], Malaysia (35.4%, n = 96) [26], and Nepal (11.4%, n = 123) [33]. Although this report was relatively lower compared to studies done in Thailand (72%, n = 100) [34], Laos (87.75%, n = 49) [35], and different reports in Malaysia (64.6% n = 82 and 87.5% n = 72) [26,36]. Prevalence of *Salmonella* contamination among different countries might be affected by differences in sampling procedures, sample types, and *Salmonella* detection and isolation techniques [37].

**Table 2:** *Salmonella-*positive retail meat samples from different animal origins and meat types. The number and percentage of samples found positive with *Salmonella* based on the food matrix using putative culture positives, TEPs I-III and overall positives from TEPs I and II. Short description regarding the animal origins, treatment, and packaging of meat sample weres also included.

Meat Samples	Meat type	Animal-origin	Treatment/Packaging	Number of samples	XLD/BG Agar plate Putative *Salmonella*	TEP I	TEP II	TEP III	Overall (Percentage)
Minced beef	Raw	Cow	Minced, no packaging	90	68	75	67	59	78 (86.67)
Chicken wings	Raw	Chicken	Chopped, no packaging	94	66	75	61	55	78 (82.98)
Ground pork	Raw	Pig	Ground, no packaging	90	72	75	64	59	79 (87.78)
Bacon	Processed	Pig	Minced, no packaging, additives, and preservatives, sometimes packaged in plastic	90	50	53	46	41	56 (62.22)
Burger	Processed	Cow	Ground, heat treatment, additives and preservatives, packaged in plastic	70	18	23	14	9	26 (37.14)
Filipino-style luncheon meat	Processed	Pig	Ground, heat treatment, additives and preservatives, packaged in aluminum foil or plastic	90	8	19	5	5	23 (25.56)
Ham	Processed	Pig	Sliced, heat treatment, additives, and preservatives sometimes packaged in plastic	85	9	21	7	6	22 (25.88)
Filipino-style sausage	Processed	Pig	Ground, additives, and preservatives, hog-casing. No packaging	96	47	50	42	40	52 (54.17)
*Salami*	Processed	Pig	Ground, additives, and preservatives, cellulose-casing. No packaging	15	4	1	0	0	1 (6.67)
Total				720	342	392	306	274	415 (57.64)

Ground pork (87.78%) was found to have the highest prevalence of *Salmonella*-contamination, followed by minced beef (86.67%), chicken wings (82.98%), bacon (62.22%), Filipino-style sausage (54.17%), burger (37.14%), Filipino-style luncheon meat (25.88%), ham (25.56%), and salami (6.67%) (Table 2). Significantly higher number of *Salmonella-*contaminated samples were observed in raw meat (*p =* 0.00001) (85.77%, n = 235) compared to processed meat samples (40.36%, n = 180). High prevalence of *Salmonella* contamination in ground pork can be associated with high frequency (45%, n = 240) of *Salmonella* found in slaughtered swine from different Metro Manila abattoirs [14,15]. Data regarding *Salmonella* frequencies in chicken and cows from slaughterhouses and farms are currently lacking. Significantly higher *Salmonella* contamination rates in raw meat compared to processed meat can be attributed to the absence of interventions which could regulate microbial growth [38]. Also, manual meat handling such as grinding may contribute to an increase of *S*. *enterica* counts in raw meat [39]. Cross-contamination of microbes in raw meat is highly probable since they were often presented and sold without packaging. Even in the presence of additives and preservatives, bacon and Filipino style sausage were observed to have a relatively higher percentage of *Salmonella* contamination (>50%) compared to other processed meat products (<38%). We observed that these meat products were processed directly in wet markets, and without heat treatment. *Salmonella* contamination in Filipino-style sausage may also come from connective and muscle tissues of the animal’s gut which serve as their natural casing [38]. Ham, Filipino-style luncheon meat, and salami had undergone heat and chemical treatments [40–44] and were packaged with either plastic or aluminum foil, hence, a lower rate of *Salmonella* contamination in these products was expected. But on several occasions, we observed that some vendors opened packaged meat products and sold them separately. The presence of *Salmonella* in ham, Filipino-style luncheon meat, and salami might also be associated with failure of treatment or cross-contamination during the manufacturing and retailing stages [45–47], however, a more elaborate study must be performed to prove this hypothesis.

We found considerable variation (5–76.25%) of *Salmonella-*contamination rate among different wet markets (Table 3), even though the handling procedures of retail meat products and environmental conditions were almost similar in all visited wet markets (S1 Appendix). Specifically, practices of meat being displayed by either hanging or lying on the counter without any covering for several hours, exposure to ambient temperature, excess moisture, and handled using bare hands made the retail meat highly vulnerable to microbial population growth and further microbial cross-contamination. Remarkably, we only found four (5%) *Salmonella* contaminated meat products (Filipino style sausages) at Quezon City District IV wet market, even in the presence of poultry evisceration activities near the meat retail area. We also noticed that the meat display counters in Makati and Paranaque wet markets were relatively drier and cleaner compared to other wet markets, and their meat counters were made up of metal or smooth concrete finishing, still, *Salmonella-*contamination rates in these markets were high (71.25% and 66.25%, respectively). Further investigation of other hygienic parameters in the wet market, such as water, equipment, vendors, and potentially other animal vectors must be performed to determine the important factors that affect *Salmonella* prevalence in wet markets [48]. Initial *Salmonella* contamination of animals from farms, slaughterhouses, and distribution may also have affected the different rates of *Salmonella-*contamination in wet markets. In previous *Salmonella* prevalence studies in abattoirs, varying *Salmonella* frequencies in slaughtered swine were observed in different slaughterhouses in Metro Manila [14,15]. We strongly suggest implementing strict hazard analysis and critical control point (HACCP) practices in slaughterhouses and transport. Hygienic practices and proper product segregation must be ensured in wet markets to prevent contamination of *Salmonella* to other fresh produce such as fruits and vegetables. Meat consumers must cook their meat thoroughly before consumption to kill microbial pathogens. Also, consumers must be cautious in handling and preparing meat to prevent cross-contamination in the kitchen and spread the pathogens to ready-to-eat food.

**Table 3:** *Salmonella-*positive retail meat samples from nine wet markets in Metro Manila. The number and percentage of samples found positive with *Salmonella* based on the wet market using putative culture positives, results of TEPs I-III and overall positives using results from TEP I and II.

Wet Markets	Number of samples	XLD/BG Agar plate Putative *Salmonella*	TEP I	TEP II	TEP III	Overall (Percentage)
Makati	80	38	53	36	35	57 (71.25)
Malabon	80	39	49	38	37	53 (66.25)
Mandaluyong	80	45	53	40	32	61 (76.25)
Manila	80	41	34	32	30	36 (45.00)
Muntinlupa	80	44	58	42	39	59 (73.75)
Paranaque	80	45	53	42	42	53 (66.25)
Pasig	80	46	46	39	24	48 (60.00)
Quezon City District IV	80	10	4	3	3	4 (5.00)
Quezon City District V	80	34	42	34	32	44 (55.00)
Total	**720**	**342**	**392**	**306**	**274**	**415 (57.64)**

### *Salmonella* serogroup and serovar distribution in retail meat

**Fig 1:**
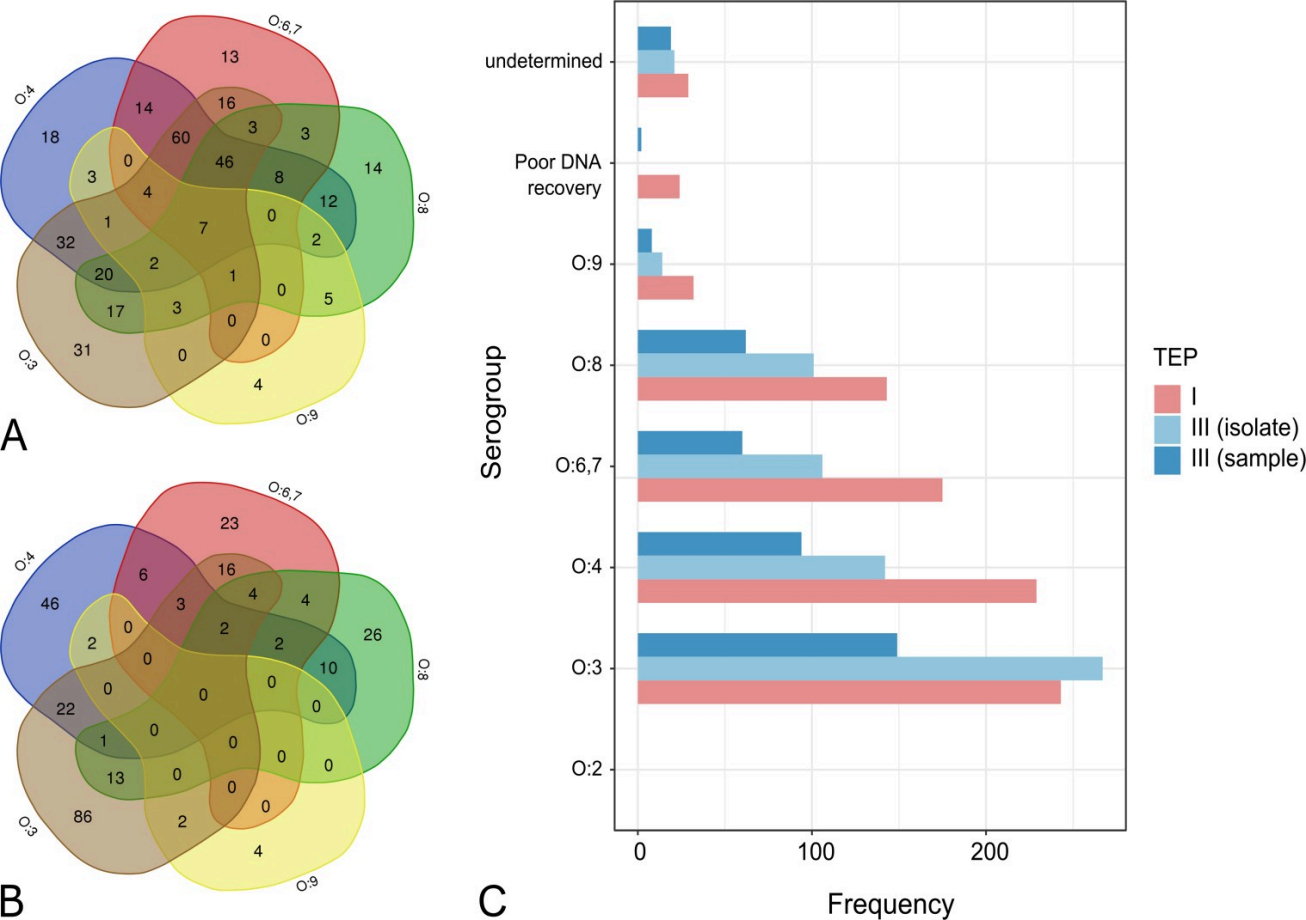
*Salmonella* serogroup frequencies in retail meat samples. 1A: Venn diagram of *Salmonella* serogroups acquired from each sample using TEP I. 1B: Venn diagram of *Salmonella* serogroups acquired from each sample using TEP III. 1C: Frequencies of *Salmonella* serogroups detected using DNA extracted in TEP I and TEP III (per isolate and per sample). Venn diagrams were created using Bioinformatics & Evolutionary Genomics webpage (URL: http://bioinformatics.psb.ugent.be/webtools/Venn/).

*Salmonella-*positive DNA extracted using TEP I (n = 392 meat samples) and TEP III (n = 641 *Salmonella* isolates from 274 meat samples) were characterized using the molecular serogrouping assay. Most meat samples were contaminated with more than one *Salmonella* serogroups using DNA from TEP I (Fig 1A; 73.47%). Since multiple putative *Salmonella* colonies (both from XLD and BG agars, if present) were isolated per samples, contamination with more than one serogroup per sample was also observed but to a lesser quantity (Fig 1B; 31.75%). These results were expected since DNA from TEP I was extracted from *Salmonella* selective enrichment broth and will likely contain mixed groups of *Salmonella*, while DNA from TEP III was isolated and purified from a single colony. Multiple *Salmonella* serogroup contaminations were also observed in slaughtered pigs from Metro Manila [14]. Detection of the same *Salmonella* serogroups from multiple isolates was also observed in several samples, hence, frequencies of *Salmonella* serogroup from TEP III (isolates) were 1.5–1.8 times higher than TEP III (samples) (Fig 1C). The *Salmonella* serogroup data produced from TEP I provide a more comprehensive overview of serogroup distribution in retail meat and the presence of mixed *Salmonella* serotype contamination. A possible underestimation of samples containing multiple serogroup contamination may have occurred since the primer pairs used in this study was limited to the detection of six *Salmonella* serogroups.

*Salmonella* serogroup O:3 was the most frequently detected serogroup in meat samples, followed by serogroup O:4 using both TEP I and TEP III. Frequencies of serogroup O:6,7 were relatively higher than O:8 using TEP I, but their frequencies were almost the same using TEP III (per sample and per isolate). The number of *Salmonella* serogroup O:9 and undetermined serogroups was almost similar in TEP I, but relatively higher frequencies of undetermined serogroup compared to serogroup O:9 were found in TEP III (per samples and per isolate). *Salmonella* serogroup O:2 was not detected in any meat sample. There was a portion of samples that did not exhibit amplification band on the internal amplification control in O-serogrouping assay likely due to either poor DNA recovery or PCR inhibitors. Previous *Salmonella* studies in Metro Manila demonstrated that *Salmonella* serogroup O:3 was the most dominant serogroup detected in raw and processed meat (78.57%) from wet markets [13], and slaughtered swine (75%) from slaughterhouses [15]. However, Ng & Rivera [14] found that serogroup O:4 (73%) was the most frequently isolated serogroup in slaughtered swine in abattoirs followed by serogroup O:3 (24%). Reports of Calayag et al. and Soguilon et al. accounted for the distribution of *Salmonella* serogroups per sample, while Ng and Rivera reported frequencies of *Salmonella* serogroups per isolate.

We detected 29 *Salmonella* band patterns that correspond to putative *Salmonella* serotypes (Table 4). Among them are 17 band patterns that correspond to a certain *Salmonella* serotype, namely, *S*. Kentucky, *S*. Saintpaul, *S*. Stanley, *S*. Typhimurium, *S*. Livingstone, *S*. Schwarzengrund, *S*. Weltevreden, *S*. Enteritidis, *S*. Virchow, *S*. Heidelberg, *S*. 4,[5],12:i:- (monophasic *S*. Typhimurium), *S*. Agama, *S*. Augustenborg, *S*. Mbandaka, *S*. Rechovot, *S*. Newlands, and *S*. Podiensis. Six band patterns corresponded to two serotypes since the O-serogrouping primers for O:3 detected both O:3,10 (*S*. Anatum, *S*. Meleagridis, *S*. Nyborg, *S*. Yeerongpilly) and O:1,3,19 (*S*. Hayindogo, *S*. Calabar, *S*. Sanktmarx, *S*. Taksony), and primer for serogroup O:8 detected both O:6,8 (*S*. Newport, *S*. Chomedey) and O:8,20 (*S*. Bardo, *S*. Glostrup) (Table 4). Serological serotyping of band patterns: *S*. 3:e,h:1,6, *S*. 8:e,h:1,2, and *S*: 3:eh:l,w resulted in *S*. Anatum, *S*. Newport, and *S*. Meleagridis, respectively. The serovar identities of the remaining six band patterns need further confirmation since the primers that target the missing somatic or flagellar antigens were not included in this study. The putative serovar identities of 6,7:g:-, 3:g:-, -:b:enx, 4:g:1,2, and 3:-:1,6, were designated as *S*. Rissen, *S*. Senftenberg, *S*. Hvitingfoss, *S*. Agona, and *S*. London, respectively, since they had been previously reported in the Philippines [49–51]. Further analyses of these band patterns together with *S*. 4:g:- must be performed to confirm serovar identity. Isolates with multiple band patterns and/or unknown serovar identities were observed in more than 100 meat samples. Further purification of the samples must be done and more primers that target different somatic and flagellar antigens must be tested to unravel the identities of these *Salmonella* isolates. This is the first study to report *Salmonella* serovars *S*. Agama, *S*. Livingstone, *S*. Meleagridis, *S*. Schwarzengrund, *S*. Gabon, *S*. 1,4,[5],12:i:-, *S*. Augustenborg, *S*. Rechovot, *S*. Newlands and *S*. Podiensis in the Philippines. Sixteen isolates with distinct *Salmonella* band patterns were sent to ARSRL for serological sequencing (Table 4), only one *Salmonella* isolate with band pattern *S*. 6,7:r:1,2 (*S*. Virchow) did not match with the serotype identity from serological testing (*S*. Gabon; band pattern: 6,7:l,w:1,2). This disagreement may have arisen from the primer pair targeting *fliC-r* gene. It was reported that this primer pair had a 99.5% specificity [12]. Hence, it is suggested to design a more specific primer pair that targets *fliC-r* gene. It is also possible that the gene that expresses the flagellar phase I (*l*,*w*) antigen was present in the isolate, but not detected due to the limited number of primers pairs used in the study. As observed in the serogroup data, contamination with multiple *Salmonella* serovars per sample was also observed. In one chicken sample, four *Salmonella* isolates were identified as *S*. Schwarzengrund, *S*. Livingstone, *S*. Rissen, and *S*. Anatum, while three *Salmonella* isolates from a burger sample were identified as *S*. Augustenborg, *S*. Kentucky and *S*. Typhimurium. More evidence of multiple *Salmonella* serovar contamination can be discovered when more primers that targeted other somatic and flagellar antigens are used in the future.

**Table 4:** Frequencies of *Salmonella* serovars in retail meat. Frequencies of *Salmonella* band patterns and their putative *Salmonella* serovar identities in retail meat that originated from cow, chicken, and pork. Results of serological serotyping of 16 *Salmonella* isolates from ARSRL were also included. Some band patterns have more than one possible identity.

*Salmonella* banding patterns	Source of uncertainty	Putative *Salmonella enterica* serotype	Serological serotyping	Animal Origin			
				Cow	Chicken	Pig	Total
3:e,h:1,6	Somatic antigen (O:3)	Anatum/Hayindogo	Anatum	14 (30)	14 (21)	52 (81)	80 (132)
6,7:g:-	Flagellar I and II antigens (-)	Rissen^1^	Rissen	8 (13)	2 (3)	32 (52)	42 (68)
8:i:z6	Confirmed	Kentucky	Kentucky	5 (6)	24 (38)	2 (2)	31 (46)
4:g:-	Flagellar I and II antigens (-)	Essen^2^	not tested	2 (2)	2 (2)	21 (30)	25 (34)
4:e,h:1,2	Confirmed	Saintpaul	Saintpaul	14 (16)	0	9 (12)	23 (28)
8:e,h:1,2	Somatic antigen (O:8)	Newport/Bardo	Newport	7 (9)	3 (5)	6 (11)	16 (25)
4:d:1,2	Confirmed	Stanley	Stanley	3 (3)	4 (8)	9 (14)	16 (25)
3:-:1,6	Flagellar I antigens (-)	London^3^	not tested	1 (2)	0	12 (16)	13 (18)
3:g:-	Flagellar I and II antigens (-)	Senftenberg^4^	Senftenberg	1 (2)	1 (1)	7 (13)	9 (16)
-:b:enx	Somatic antigen (-)	Hvittingfoss^5^	Hvittingfoss	4 (4)	0	4 (5)	8 (9)
4:i:1,2	Confirmed	Typhimurium	Typhimurium	4 (4)	0	3 (3)	7 (7)
6,7:d:lw	Confirmed	Livingstone	Livingstone	1 (1)	3 (5)	2 (4)	6 (10)
4:d:1,7	Confirmed	Schwarzengrund	Schwarzengrund	0	4 (6)	2 (2)	6 (8)
4:i:-	Confirmed	Monophasic *S*. Typhimurium	not tested	0	2 (2)	4 (9)	6 (11)
3:r:z6	Confirmed	Weltevreden	Weltevreden	1 (2)	1 (1)	3 (4)	5 (7)
6,7:r:1,2	Confirmed	Virchow	Gabon	1 (1)	3 (5)	0	4 (6)
9:g:- (sdf-I +)	Confirmed	Enteritidis	Enteritidis	0	2 (4)	2 (2)	4 (6)
3:eh:lw	Somatic antigen (O:3)	Meleagridis/Calabar	Meleagridis	0	0	4 (6)	4 (6)
4:r:1,2	Confirmed	Heidelberg	not tested	0	1 (3)	1 (1)	2 (4)
4:i:1,6	Confirmed	Agama	not tested	0	2 (2)	0	2 (2)
3:eh:1,7	Somatic antigen (O:3)	Nyborg/Sanktmarx	not tested	1 (1)	0	1 (1)	2 (2)
6,7:i:1,2	Confirmed	Augustenborg	not tested	1 (2)	1 (2)	0	2 (4)
4:g:1,2	Flagellar I and II antigens	Agona^6^	not tested	0	0	1 (1)	1 (1)
6,7:z10:enz15	Confirmed	Mbandaka	Mbandaka	0	0	1 (1)	1 (1)
8:eh:z6	Confirmed	Rechovot	not tested	1 (1)	0	0	1 (1)
8:z10:enz15	Somatic antigen (O:8)	Chomedey/Glostrup	not tested	0	0	1 (1)	1 (1)
3:eh:enx	Confirmed	Newlands	not tested	1 (1)	0	1 (1)	1 (1)
3:z10:enx	Confirmed	Podiensis	not tested	0	0	1 (1)	1 (1)
3:i:z6	Somatic antigen (O:3)	Yeerongpilly/Taksony	not tested	0	0	1 (1)	1 (1)
Multiple/Incomplete annotation							
O:4	Unknown			7 (8)	4 (4)	8 (10)	19 (22)
O:6,7	Unknown			4 (4)	4 (4)	6 (7)	14 (15)
O:8	Unknown			9 (15)	4 (4)	6 (7)	19 (26)
O:9	Unknown			2 (5)	0	2 (3)	4 (8)
O:3	Unknown			16 (17)	7 (7)	36 (59)	59 (83)
Unknown	Unknown			3 (4)	8 (12)	5 (8)	0

^1^Other possible serovar identities: *S*. Eingedi/ *S*. Montevideo/ *S*. II 6,7:g,m,s,t:z39/*S*. II 6,7:g,[m],s,t:[z42]/ *S*. Othmarschen/ *S*. Plumaugat/ *S*. Riggil/ *S*. IV 6,7:g,z51:-/ *S*. Haelsingborg/ *S*. Oakey/ *S*. II 6,7:m,t:-.

^2^*S*. Essen/ *S*. California/ *S*. Budapest/ *S*. Banana/ *S*. II 1,4,12,[27]:g,[m],t:[1,5]/ *S*. II 4,12:g,m,t:z39/ *S*. II 4,12:g,z62:-.

^3^*S*. Ikayi/ *S*. Regent/ *S*. Kainji/ *S*. Harleystreet/ *S*. Ratchaburi/ *S*. Albertslund/ *S*. Bida/ *S*. Winterthur.

^**4**^*S*. Regent/ *S*. Suberu/ *S*. Amsterdam/ *S*. II 3,{10}{15}:g,m,s,t:[1,5]/ *S*. Cannstatt/ *S*. Westhampton/ *S*. II 3,10:g,t:-/ *S*. Southbank/ *S*. Kouka.

^**5**^*S*. Abony/ *S*. II 1,4,[5],12,[27]:b:[e,n,x]/ *S*. Lockleaze/ *S*. Konstanz/ *S*. Gatuni/ *S*. II 1,9,12:b: [e,n,x]/ *S*. Worb/ *S*. II 9,46:b: [e,n,x]/ *S*. Benfica/ *S*. II 3,10:b: [e,n,x]/ *S*. Tambacounda/ *S*. VI 11:b: [e,n,x]/ *S*. Vaertan/Ullevi/ *S*. IIIb (6),14:b: [e,n,x]/ *S*. II 16:b: [e,n,x]/ *S*. Mattenhof/ *S*. Minnesota/ *S*. Soumbedioune/II 28:b: [e,n,x]/ *S*. Urbana/ *S*. Johannesburg/ *S*. Elbeuf/ *S*. Flottbek/ *S*. Tonev.

^*6*^*S*. *Agona*/ *S*.Derby/ *S*.Hato/ *S*. Kingston.

The most frequently detected serovar in chicken was *S*. Kentucky, while *S*. Anatum was the most frequently detected in meat originating from pigs and cows. In addition to *S*. Anatum, *S*. Saintpaul had the same detection frequency in minced beef and burger. In the Philippines, *Salmonella* Kentucky was isolated from cloacal swabs of healthy layer chickens in San Jose, Batangas in 2011 [51]. In other countries, *S*. Kentucky was the most commonly reported serovar for poultry in Canada and the US, ground chicken and broilers in the US, and broiler meat in 11 European countries in 2007 [3]. *Salmonella* Kentucky was also the most commonly isolated serovar in retail chicken meat in Vietnam [29], and Trinidad [52]. *Salmonella* Anatum was previously reported in mesenteric lymph nodes of asymptomatic cattle in Laguna and Batangas [53], and bile specimens of healthy cattle [54] in the Philippines. In other countries, *S*. Anatum was included in ten most frequently isolated serovars in cattle in the United Kingdom, US and Tunisia, in bovine meat in Canada, ground beef in the US, included in the seven most common serovars isolated in pigs and livestock in the United Kingdom in 2007, and most frequently isolated serovar in pork within Thailand for 2003 [55]. This serovar was also the most prevalent in cattle and swine from Vietnam in 2004 [56]. *Salmonella* Saintpaul was previously detected in asymptomatic cattle from Laguna and Batangas, Philippines [53]. And frequently isolated in apparently healthy cattle in Australasia (5.4%) and Africa (2.1%) [57]. Other *Salmonella* serovars such as *S*. Newport, *S*. Stanley, *S*. Derby, *S*. Weltevreden, and *S*. Enteritidis were previously reported in animal and retail meat [15,31,53,54], while *S*. Senftenberg and *S*. Hvittingfoss were reported in peanut butter products in the Philippines.

These detected serovars can pose risks to retail meat vendors and consumers through unhygienic handling, cross-contamination with ready-to-eat food such as fresh produce, and consumption of poorly cooked meat. All previously reported serovars in the Philippines had been isolated in human clinical cases in the country. Although *S*. Anatum, *S*. Kentucky, and *S*. Saintpaul were the most frequently detected serovars in retail meat, reports regarding their incidence rates among submitted clinical isolates from 2004–2018 were very low. The incidence rate of *S*. Anatum was 0.54% (n = 13) in clinical isolates in the Philippines from 2004–2018 [58], while the incidence rates of *S*. Kentucky and *S*. Saintpaul were both 0.26% (n = 2) from 2014–2018 [59,60–63]. These serovars may possess low virulence and induce moderate symptoms in humans, wherein patients no longer seek medical care. Hence, clinical cases caused by these serovars may be underreported. It was estimated that the true incidence of salmonellosis is at least 29-fold greater than the number of reported cases [64]. The most commonly reported non-typhoidal *Salmonella* serovars in human clinical cases in the Philippines are *S*. Enteritidis and *S*. Typhimurium from 2003–2018 [49,50,59,65], however, they ranked 11^th^ and 10^th^ frequently isolated *Salmonella* serovars in this study. Low prevalence rates of these serovars in meat samples suggest that their mode of transmission may come from other significant reservoirs, which have not yet been fully studied. It is recommended to perform more screening and serotype distribution studies in live and slaughtered animals, meat and other food sources (eggs, fresh produce, milk, etc.), and environmental samples in different parts of the country to obtain a more comprehensive picture of the extent of atypical *Salmonella* serovars associated with food contamination. Another hypothesis is that these serovars are more virulent to humans compared to other predominant *Salmonella* serovars, which resulted in infections that required medical attention to patients and were followed up by strain isolation and incidence reports. In contrast to this findings, most commonly reported serovars in human clinical cases in the United States (*S*. Enteritidis, *S*. Typhimurium, *S*. Newport, *S*. Javiana, *S*. I 4,[5],12:I:-, and *S*. Muenchen) were also reported as commonly isolated *Salmonella* serotypes (except for *S*. Javiana and *S*. Muenchen) in meat and meat-producing animals in the US [64].

Published reports *of Salmonella* serogroup and serovar distribution in animal, food, and environmental samples are limited and outdated in the Philippines. Studies that characterized *Salmonella* up to the serovar level were performed mostly by researchers from different academic institutions more than a decade ago [31,54,55]. Moreover, the determination of *Salmonella* serovar distribution in the food production chain is often neglected. Reports from the Philippine Department of Agriculture (DA)-mandated laboratories regarding *Salmonella* were focused on faster detection of the pathogen in animal, food, and environmental samples, and determination of their antimicrobial susceptibility profiles (personal communication with the National Meat Inspection Service, Philippines). Thus, this study offers a more recent overview of prevailing *Salmonella* serogroups and serovars present in retail meat that originates from cattle, poultry, and swine. Surveillance of *Salmonella* and its serovars in different reservoirs is important in source tracking and attribution, mitigation, and control of *Salmonella*-related outbreaks. In Denmark, *Salmonella* surveillance data were used in their targeted national control program incorporating HACCP, a systemic preventive approach to food safety, wherein key steps in the food to fork chain were identified for interventions that have an impact on reduction (or elimination) of food safety hazards. This control program caused a significant reduction in human salmonellosis in Denmark. The prevalence of salmonellosis in the broiler, pork, and eggs was also reduced by 95%, 85%, and 75%, respectively [66].

## Conclusion

The molecular identification and characterization of *Salmonella* provide baseline information regarding its prevalence and serovar distribution in food samples originated from chicken, pigs, and cows. The PCR-based assay that targeted *invA* gene revealed that 415 (57.64%) retail meat samples were contaminated with *Salmonella*, while molecular serotyping showed that most retail meats were contaminated with multiple *Salmonella* serovars. We have demonstrated a faster means of *Salmonella* detection in food using the TEP I method, which confirmed the pathogen within 2 days compared to the traditional culture technique that requires 5–7 days, and higher sensitivity of *Salmonella* detection when the protocols TEP I and II were combined. Classical serotyping of *Salmonella* in animal and environmental samples are not routinely performed in the Philippines since the technique is expensive, time-consuming, and requires skilled technicians. Thus, there is a need for the development or adaptation of rapid, specific, sensitive, less costly, and high throughput techniques. This study adapted previously developed primers to characterize the serogroups and serovars of *Salmonella* isolates from retail meat using multiplex PCR. We detected five serogroups and at least 29 band patterns that lead to putative *Salmonella s*erovar identities. We also found evidence of multiple *Salmonella* serogroup and serovar contaminations per sample using TEP I and TEP III. It is highly recommended to adapt or design more primers that target somatic and flagellar genes to determine the identities of unknown isolates. This molecular *Salmonella* serotyping assay may be adapted by laboratories from health and agricultural departments, particularly for developing countries, as an alternative approach to classical serotyping techniques to increase the *Salmonella* surveillance data in these regions.

This surveillance data is important in the development of *Salmonella* control strategy, intervention, and prevention schemes. We determined the most predominant *Salmonella* serovars in retail meat originating from were *S*. 3:e,h:1,6 (putative identity: *S*. Anatum) from pig, *S*. 3:e,h:1,6 (putative identity: *S*. Anatum), and *S*. 4:e,h:1,2 (putative identity: *S*. Saintpaul) from cow, and *S*. 8:i:z6 (putative identity: *S*. Kentucky) from chicken. On the other hand, we observed the low prevalence of *S*. Typhimurium and *S*. Enteritidis in retail meat even when they are the most commonly reported serovars in human clinical samples. This is also the first study to report *Salmonella* serovars *S*. Agama, *S*. Livingstone, *S*. Meleagridis, *S*. Schwarzengrund, monophasic *S*. Typhimurium, *S*. Augustenbirg, *S*. Rechovot, *S*. Newlands, *S*. Podiensis, and *S*. Gabon in the Philippines. It is proposed to include non-human and non-clinical isolates in routine *Salmonella* serotyping, particularly in the food production chain, to track the sources of *Salmonella* contamination in food and future outbreaks in humans and/or animals. Investigation of the antimicrobial susceptibility of *Salmonella* isolates acquired from this study shall be performed in future studies to identify emerging resistant strains circulating in foods of animal origin, in particular from cows, swine, and poultry.

## Supporting information

S1 Appendix Wet market assessment sheets. Guide questions used to assess the hygienic conditions of each wet markets in Metro Manila. The name of wet markets was concealed. “*Tocino”*, *“Embutido”* and “*Longganisa”* are Filipino terms for bacon, Filipino style meatloaf, and Filipino style sausage.
(DOCX)
