# Supplementary material for: PCR-based detection and serovar identification of Salmonella in retail meat collected from wet markets in Metro Manila, Philippines
Source: PLoS One. 2020 Sep 30;15(9):e0239457. doi: 10.1371/journal.pone.0239457 (PMC7526908; doi:10.1371/journal.pone.0239457)
Supplement: S1 Appendix — Guide questions used to assess the hygienic conditions of each wet markets in Metro Manila. The name of wet markets was concealed. “Tocino”, “Embutido” and “Longganisa” are Filipino terms for bacon, Filipino style meatloaf, and Filipino style sausage. (DOCX) [file pone.0239457.s001.docx]

**S1 Appendix. Wet Market Assessment sheets**. Guide questions used to assess the hygienic conditions of each wet markets in Metro Manila. The name of wet markets was concealed. “*Tocino”, “Embutido”* and “*Longganisa”* are Filipino terms for bacon, Filipino style meatloaf, and Filipino style sausage.

Market: xxxx City: Quezon City, District IV

Date: 22 May 2017

Time of inspection: 9:15 am

|  | **1** | **2** | **3** | **4** | **5** |
| --- | --- | --- | --- | --- | --- |
| **A. Lighting** |  |  |  | **X** |  |
| **B. Ventilation** |  |  | **X** |  |  |
| **C. Floorings** |  |  |  |  |  |
| Finished? |  | **X** |  |  |  |
| Moisture (Dry 🡪 Wet) |  | **X** |  |  |  |
| Cleanliness |  |  |  | **X** |  |
| **D. Display Tables** |  |  |  |  |  |
| Finished? |  |  |  | **X** |  |
| Smooth surface |  |  |  | **X** |  |
| Drains? |  |  |  | **X** |  |
| Elevation |  |  | **X** |  |  |
| Cleanliness |  |  |  | **X** |  |
| Moisture (*ice/water/blood*) |  |  |  | **X** |  |
| **E. Vendors/Food handlers** |  |  |  |  |  |
| Proper attire |  |  | **X** |  |  |
| Transact while selling |  |  | **X** |  |  |
| **F. Equipment (*cleanliness*)** |  |  |  |  |  |
| Knife |  |  | **X** |  |  |
| Grinder |  |  | **X** |  |  |
| Chopping board |  |  | **X** |  |  |
| **G. Zoning for the whole market** |  |  |  |  |  |
| Meat and meat food  Products |  |  | **X** |  |  |
| Chicken (isolated) |  |  | **X** |  |  |
| Pork |  |  | **X** |  |  |
| Beef |  |  | **X** |  |  |
| **H. Pest** |  |  |  |  |  |
| Flies (absence 🡪 presence) |  |  |  | **X** |  |
| Cockroaches (absence 🡪  presence) |  |  |  | **X** |  |
| Rats |  |  |  |  |  |
| **I. Cleanliness** |  |  |  |  |  |
| Waste segregation |  |  |  | **X** |  |
| Trash can |  |  |  | **X** |  |
| Trash |  |  |  | **X** |  |
| Odor |  |  | **X** |  |  |
| **J. Cooling system** |  |  |  |  |  |
| Refrigerator/Chiller |  |  |  |  | **X** |
| Ice |  |  |  |  | **X** |
| **M. Sewage (presence)** |  |  |  |  |  |
| Clean |  |  | **X** |  |  |
| **N. Meat** |  |  |  |  |  |
| Handling |  |  | **X** |  |  |
| Freshness |  | **X** |  |  |  |
| **O. Meat stalls** |  |  |  |  |  |
| Separate from fish stalls |  | **X** |  |  |  |
| Evisceration, skinning |  |  |  | **X** |  |
| Chopping blocks, easily  cleaned, free of cracks and  crevices |  |  | **X** |  |  |
| Hang in hanging rails, not lying  on the counter |  |  |  | **X** |  |
| Clean |  |  | **X** |  |  |
| Free for vermin |  |  |  |  |  |
| Processing of meat (tocino,  longganisa) | Present, tocino, longganisa near dry goods | | | | |

1 – Very good 4 - Bad

2 – Good 5- Worst

3 – Neutral

Remarks:

- Some isolated meat stalls are near cooked food and fresh produce.
- The evisceration of chicken is observed within the market
- Pans for lying meat are absent

Market: xxx City: Malabon

Date: 22 May 2017

Time of inspection: 10:40 am

|  | **1** | **2** | **3** | **4** | **5** |
| --- | --- | --- | --- | --- | --- |
| **A. Lighting** |  |  |  | **X** |  |
| **B. Ventilation** |  |  | **X** |  |  |
| **C. Floorings** |  |  |  |  |  |
| Finished? |  | **X** |  |  |  |
| Moisture (Dry 🡪 Wet) |  | **X** |  |  |  |
| Cleanliness |  |  |  | **X** |  |
| **D. Display Tables** |  |  |  |  |  |
| Finished? |  | **X** |  |  |  |
| Smooth surface |  | **X** |  |  |  |
| Drains? |  |  | **X** |  |  |
| Elevation |  |  | **X** |  |  |
| Cleanliness |  |  |  |  | **X** |
| Moisture (*ice/water/blood*) |  |  |  |  | **X** |
| **E. Vendors/Food handlers** |  |  |  |  |  |
| Proper attire |  |  |  | **X** |  |
| Transact while selling |  |  | **X** |  |  |
| **F. Equipment (*cleanliness*)** |  |  |  |  |  |
| Knife |  |  | **X** |  |  |
| Grinder |  |  | **X** |  |  |
| Chopping board |  |  | **X** |  |  |
| **G. Zoning for whole market** |  |  |  |  |  |
| Meat and meat food  Products |  | **X** |  |  |  |
| Chicken (isolated) |  | **X** |  |  |  |
| Pork |  | **X** |  |  |  |
| Beef |  | **X** |  |  |  |
| **H. Pest** |  |  |  |  |  |
| Flies (absence 🡪 presence) |  |  |  |  | **X** |
| Cockroaches (absence 🡪  presence) |  |  |  |  |  |
| Rats |  |  |  |  |  |
| **I. Cleanliness** |  |  |  |  |  |
| Waste segregation |  |  |  |  | **X** |
| Trash can |  |  |  |  | **X** |
| Trash |  |  |  |  | **X** |
| Odor |  |  |  |  | **X** |
| **J. Cooling system** |  |  |  |  |  |
| Refrigerator/Chiller |  |  |  |  | **X** |
| Ice |  |  |  |  | **X** |
| **M. Sewage (presence)** |  |  |  |  |  |
| Clean |  |  | **X** |  |  |
| **N. Meat** |  |  |  |  |  |
| Handling |  |  | **X** |  |  |
| Freshness |  | **X** |  |  |  |
| **O. Meat stalls** |  |  |  |  |  |
| Separate from fish stalls |  | **X** |  |  |  |
| Evisceration, skinning |  | **X** |  |  |  |
| Chopping blocks, easily  cleaned, free of cracks and  crevices |  |  | **X** |  |  |
| Hang in hanging rails, not lying  on the counter |  | **X** |  |  |  |
| Clean |  |  |  |  | **X** |
| Free for vermin |  |  |  |  |  |
| Processing of meat (tocino,  longganisa) | Present: Longganisa, tocino, barbeque and marinated chicken | | | | |

1 – Very good 4 - Bad

2 – Good 5- Worst

3 – Neutral

Remarks:

- Ice present in some meat counter
- Some of the vendors are not wearing shirts.

Markets: xxx City: Makati

Date: 20 May 2017

Time of inspection: 10:30 am

|  | **1** | **2** | **3** | **4** | **5** |
| --- | --- | --- | --- | --- | --- |
| **A. Lighting** | **X** |  |  |  |  |
| **B. Ventilation** | **X** |  |  |  |  |
| **C. Floorings** |  |  |  |  |  |
| Finished? | **X** |  |  |  |  |
| Moisture (Dry 🡪 Wet) | **X** |  |  |  |  |
| Cleanliness | **X** |  |  |  |  |
| **D. Display Tables** |  |  |  |  |  |
| Finished? | **X** |  |  |  |  |
| Smooth surface | **X** |  |  |  |  |
| Drains? | **X** |  |  |  |  |
| Elevation | **X** |  |  |  |  |
| Cleanliness | **X** |  |  |  |  |
| Moisture (*ice/water/blood*) | **X** |  |  |  |  |
| **E. Vendors/Food handlers** |  |  |  |  |  |
| Proper attire | **X** |  |  |  |  |
| Transact while selling |  |  | **X** |  |  |
| **F. Equipment (*cleanliness*)** |  |  |  |  |  |
| Knife |  |  | **X** |  |  |
| Grinder |  |  | **X** |  |  |
| Chopping board |  |  | **X** |  |  |
| **G. Zoning for whole market** |  |  |  |  |  |
| Meat and meat food  Products | **X** |  |  |  |  |
| Chicken (isolated) | **X** |  |  |  |  |
| Pork | **X** |  |  |  |  |
| Beef | **X** |  |  |  |  |
| **H. Pest** |  |  |  |  |  |
| Flies (absence 🡪 presence) |  |  | **X** |  |  |
| Cockroaches (absence 🡪  presence) |  |  |  |  |  |
| Rats |  |  |  |  |  |
| **I. Cleanliness** |  |  |  |  |  |
| Waste segregation | **X** |  |  |  |  |
| Trash can | **X** |  |  |  |  |
| Trash | **X** |  |  |  |  |
| Odor | **X** |  |  |  |  |
| **J. Cooling system** |  |  |  |  |  |
| Refrigerator/Chiller |  |  |  |  | **X** |
| Ice |  |  |  |  | **X** |
| **M. Sewage (presence)** |  |  |  |  |  |
| Clean | **X** |  |  |  |  |
| **N. Meat** |  |  |  |  |  |
| Handling |  |  | **X** |  |  |
| Freshness |  | **X** |  |  |  |
| **O. Meat stalls** |  |  |  |  |  |
| Separate from fish stalls | **X** |  |  |  |  |
| Evisceration, skinning | **X** |  |  |  |  |
| Chopping blocks, easily  cleaned, free of cracks and  crevices |  |  | **X** |  |  |
| Hang in hanging rails, not lying  on the counter |  | **X** |  |  |  |
| Clean | **X** |  |  |  |  |
| Free for vermin |  |  |  |  |  |
| Processing of meat (tocino,  longganisa) | Present: tocino, longganisa, | | | | |

1 – Very good 4 - Bad

2 – Good 5- Worst

3 – Neutral

Remarks:

- The market floor and display table are dry and clean.
- The display table is finished or made up of metal.
- Vendors wear uniform
- Meats are segregated per animal origin.

Markets: xxx City: Paranaque

Date: 20 May 2017

Time of inspection: 9:39 am

|  | **1** | **2** | **3** | **4** | **5** |
| --- | --- | --- | --- | --- | --- |
| **A. Lighting** | **X** |  |  |  |  |
| **B. Ventilation** | **X** |  |  |  |  |
| **C. Floorings** |  |  |  |  |  |
| Finished? | **X** |  |  |  |  |
| Moisture (Dry 🡪 Wet) | **X** |  |  |  |  |
| Cleanliness |  | **X** |  |  |  |
| **D. Display Tables** |  |  |  |  |  |
| Finished? | **X** |  |  |  |  |
| Smooth surface | **X** |  |  |  |  |
| Drains? | **X** |  |  |  |  |
| Elevation | **X** |  |  |  |  |
| Cleanliness | **X** |  |  |  |  |
| Moisture (*ice/water/blood*) | **X** |  |  |  |  |
| **E. Vendors/Food handlers** |  |  |  |  |  |
| Proper attire |  |  |  | **X** |  |
| Transact while selling |  |  | **X** |  |  |
| **F. Equipment (*cleanliness*)** |  |  |  |  |  |
| Knife |  |  | **X** |  |  |
| Grinder |  |  | **X** |  |  |
| Chopping board |  |  | **X** |  |  |
| **G. Zoning for the whole market** |  |  |  |  |  |
| Meat and meat food  Products |  |  |  |  | **X** |
| Chicken (isolated) |  |  |  |  | **X** |
| Pork |  |  |  |  | **X** |
| Beef |  |  |  |  | **X** |
| **H. Pest** |  |  |  |  |  |
| Flies (absence 🡪 presence) |  |  | **X** |  |  |
| Cockroaches (absence 🡪  presence) |  |  |  |  |  |
| Rats |  |  |  |  |  |
| **I. Cleanliness** |  |  |  |  |  |
| Waste segregation |  | **X** |  |  |  |
| Trash can |  |  | **X** |  |  |
| Trash |  |  |  | **X** |  |
| Odor |  | **X** |  |  |  |
| **J. Cooling system** |  |  |  |  |  |
| Refrigerator/Chiller |  |  |  |  | **X** |
| Ice |  |  |  |  | **X** |
| **M. Sewage (presence)** |  |  |  |  |  |
| Clean |  | **X** |  |  |  |
| **N. Meat** |  |  |  |  |  |
| Handling |  |  | **X** |  |  |
| Freshness |  | **X** |  |  |  |
| **O. Meat stalls** |  |  |  |  |  |
| Separate from fish stalls |  | **X** |  |  |  |
| Evisceration, skinning | **X** |  |  |  |  |
| Chopping blocks, easily  cleaned, free of cracks and  crevices |  |  | **X** |  |  |
| Hang in hanging rails, not lying  on the counter | **X** |  |  |  |  |
| Clean |  | **X** |  |  |  |
| Free for vermin |  |  |  |  |  |
| Processing of meat (tocino,  longganisa) | Present: tocino, marinated chicken, longganisa and shanghai (wrapped ground meat). | | | | |

1 – Very good 4 - Bad

2 – Good 5- Worst

3 – Neutral

Remarks:

- Meat area can be divided into 2
  - Separate meat area: clean, dry (floor and display table), no odor
  - on the road: fused with a dry area
- Meats were displayed on dry containers (pans), some with cartons.
- Most people are wearing clothes, some are observed without clothes.
- Some stalls are within meat stall area, while other meat stalls are fused with dry section.

Markets: xxx City: Muntinlupa

Date: 20 May 2017

Time of inspection: 8:30 am

|  | **1** | **2** | **3** | **4** | **5** |
| --- | --- | --- | --- | --- | --- |
| **A. Lighting** |  | **X** |  |  |  |
| **B. Ventilation** |  |  | **X** |  |  |
| **C. Floorings** |  |  |  |  |  |
| Finished? |  |  | **X** |  |  |
| Moisture (Dry 🡪 Wet) |  |  |  |  | **X** |
| Cleanliness |  |  |  |  | **X** |
| **D. Display Tables** |  |  |  |  |  |
| Finished? |  | **X** |  |  |  |
| Smooth surface |  |  | **X** |  |  |
| Drains? |  |  |  |  | **X** |
| Elevation |  | **X** |  |  |  |
| Cleanliness |  |  |  | **X** |  |
| Moisture (*ice/water/blood*) |  |  |  |  | **X** |
| **E. Vendors/Food handlers** |  |  |  |  |  |
| Proper attire |  | **X** |  |  |  |
| Transact while selling |  |  | **X** |  |  |
| **F. Equipment (*cleanliness*)** |  |  |  |  |  |
| Knife |  |  | **X** |  |  |
| Grinder |  |  | **X** |  |  |
| Chopping board |  |  | **X** |  |  |
| **G. Zoning for whole market** |  |  |  |  |  |
| Meat and meat food  Products |  |  |  |  | **X** |
| Chicken (isolated) |  |  |  |  | **X** |
| Pork |  |  |  |  | **X** |
| Beef |  |  |  |  | **X** |
| **H. Pest** |  |  |  |  |  |
| Flies (absence 🡪 presence) |  |  |  |  | **X** |
| Cockroaches (absence 🡪  presence) |  |  |  |  |  |
| Rats |  |  |  |  |  |
| **I. Cleanliness** |  |  |  |  |  |
| Waste segregation |  |  |  | **X** |  |
| Trash can |  | **X** |  |  |  |
| Trash |  | **X** |  |  |  |
| Odor |  |  |  | **X** |  |
| **J. Cooling system** |  |  |  |  |  |
| Refrigerator/Chiller |  |  |  |  | **X** |
| Ice |  |  |  |  | **X** |
| **M. Sewage (presence)** |  |  |  |  |  |
| Clean |  |  |  | **X** |  |
| **N. Meat** |  |  |  |  |  |
| Handling |  |  | **X** |  |  |
| Freshness |  | **X** |  |  |  |
| **O. Meat stalls** |  |  |  |  |  |
| Separate from fish stalls |  |  |  |  | **X** |
| Evisceration, skinning | **X** |  |  |  |  |
| Chopping blocks, easily  cleaned, free of cracks and  crevices |  |  | **X** |  |  |
| Hang in hanging rails, not lying  on the counter |  | **X** |  |  |  |
| Clean |  |  |  | **X** |  |
| Free for vermin |  |  |  |  |  |
| Processing of meat (tocino,  longganisa) | Processing not observed. | | | | |

1 – Very good 4 - Bad

2 – Good 5- Worst

3 – Neutral

Remarks:

- Meat area is not separated from fish area.
- Muddy floor.

Markets: xxx City: Mandaluyong

Date: 20 May 2017

Time of inspection: 12:00 am

|  | **1** | **2** | **3** | **4** | **5** |
| --- | --- | --- | --- | --- | --- |
| **A. Lighting** |  |  |  |  | **X** |
| **B. Ventilation** |  |  |  |  | **X** |
| **C. Floorings** |  |  |  |  |  |
| Finished? |  |  | **X** |  |  |
| Moisture (Dry 🡪 Wet) |  |  |  | **X** |  |
| Cleanliness |  |  |  |  | **X** |
| **D. Display Tables** |  |  |  |  |  |
| Finished? |  |  | **X** |  |  |
| Smooth surface |  |  |  | **X** |  |
| Drains? |  |  |  | **X** |  |
| Elevation |  | **X** |  |  |  |
| Cleanliness |  |  | **X** |  |  |
| Moisture (*ice/water/blood*) |  |  |  |  | **X** |
| **E. Vendors/Food handlers** |  |  |  |  |  |
| Proper attire |  |  | **X** |  |  |
| Transact while selling |  |  | **X** |  |  |
| **F. Equipment (*cleanliness*)** |  |  |  |  |  |
| Knife |  |  | **X** |  |  |
| Grinder |  |  | **X** |  |  |
| Chopping board |  |  | **X** |  |  |
| **G. Zoning for whole market** |  |  |  |  |  |
| Meat and meat food  Products |  | **X** |  |  |  |
| Chicken (isolated) |  | **X** |  |  |  |
| Pork |  | **X** |  |  |  |
| Beef |  | **X** |  |  |  |
| **H. Pest** |  |  |  |  |  |
| Flies (absence 🡪 presence) |  |  |  | **X** |  |
| Cockroaches (absence 🡪  presence) |  |  |  |  |  |
| Rats |  |  |  |  |  |
| **I. Cleanliness** |  |  |  |  |  |
| Waste segregation |  |  |  |  | **X** |
| Trash can |  |  |  |  | **X** |
| Trash |  |  |  |  | **X** |
| Odor |  |  |  |  | **X** |
| **J. Cooling system** |  |  |  |  |  |
| Refrigerator/Chiller |  |  | **X** |  |  |
| Ice |  |  |  |  |  |
| **M. Sewage (presence)** |  |  |  |  |  |
| Clean |  |  |  |  | **X** |
| **N. Meat** |  |  |  |  |  |
| Handling |  |  | **X** |  |  |
| Freshness |  | **X** |  |  |  |
| **O. Meat stalls** |  |  |  |  |  |
| Separate from fish stalls |  | **X** |  |  |  |
| Evisceration, skinning | **X** |  |  |  |  |
| Chopping blocks, easily  cleaned, free of cracks and  crevices |  |  | **X** |  |  |
| Hang in hanging rails, not lying  on the counter |  | **X** |  |  |  |
| Clean |  |  |  |  | **X** |
| Free for vermin |  |  |  |  |  |
| Processing of meat (tocino,  longganisa) | Mini-meat processing section within the market, near meat section | | | | |

1 – Very good 4 - Bad

2 – Good 5- Worst

3 – Neutral

Remarks:

- Fish products are isolated in meat products. However, in some areas, meat products are not properly segregated per animal origin.

Markets: xxx City: Pasig

Date: 20 May 2017

Time of inspection: 11:27 am

|  | **1** | **2** | **3** | **4** | **5** |
| --- | --- | --- | --- | --- | --- |
| **A. Lighting** |  |  |  | **X** |  |
| **B. Ventilation** |  |  | **X** |  |  |
| **C. Floorings** |  |  |  |  |  |
| Finished? |  |  | **X** |  |  |
| Moisture (Dry 🡪 Wet) |  |  |  | **X** |  |
| Cleanliness |  |  |  | **X** |  |
| **D. Display Tables** |  |  |  |  |  |
| Finished? |  | **X** |  |  |  |
| Smooth surface |  | **X** |  |  |  |
| Drains? | **X** |  |  |  |  |
| Elevation | **X** |  |  |  |  |
| Cleanliness |  |  |  | **X** |  |
| Moisture (*ice/water/blood*) |  |  |  | **X** |  |
| **E. Vendors/Food handlers** |  |  |  |  |  |
| Proper attire |  |  | **X** |  |  |
| Transact while selling |  |  | **X** |  |  |
| **F. Equipment (*cleanliness*)** |  |  |  |  |  |
| Knife |  |  | **X** |  |  |
| Grinder |  |  | **X** |  |  |
| Chopping board |  |  | **X** |  |  |
| **G. Zoning for whole market** |  |  |  |  |  |
| Meat and meat food  Products | **X** |  |  |  |  |
| Chicken (isolated) | **X** |  |  |  |  |
| Pork | **X** |  |  |  |  |
| Beef | **X** |  |  |  |  |
| **H. Pest** |  |  |  |  |  |
| Flies (absence 🡪 presence) |  |  |  | **X** |  |
| Cockroaches (absence 🡪  presence) |  |  |  |  |  |
| Rats |  |  |  |  |  |
| **I. Cleanliness** |  |  |  |  |  |
| Waste segregation |  |  |  |  | **X** |
| Trash can |  |  |  | **X** |  |
| Trash |  |  |  |  | **X** |
| Odor |  |  |  | **X** |  |
| **J. Cooling system** |  |  |  |  |  |
| Refrigerator/Chiller |  |  |  |  | **X** |
| Ice |  |  |  | **X** |  |
| **M. Sewage (presence)** |  |  |  |  |  |
| Clean |  |  | **X** |  |  |
| **N. Meat** |  |  |  |  |  |
| Handling |  |  | **X** |  |  |
| Freshness |  | **X** |  |  |  |
| **O. Meat stalls** |  |  |  |  |  |
| Separate from fish stalls | **X** |  |  |  |  |
| Evisceration, skinning | **X** |  |  |  |  |
| Chopping blocks, easily  cleaned, free of cracks and  crevices |  |  | **X** |  |  |
| Hang in hanging rails, not lying  on the counter |  | **X** |  |  |  |
| Clean |  |  |  | **X** |  |
| Free for vermin |  |  |  |  |  |
| Processing of meat (tocino,  longganisa) | Present: longganisa, tocino, and tapa were observed. Placed on covered container. | | | | |

1 – Very good 4 - Bad

2 – Good 5- Worst

3 – Neutral

Remarks:

- Ice for chicken feet
- Meat cuts and feather on the floor.
- Chicken products are displayed on metal counters, while other meat products are displayed on wooden counters.
- Meat based from animal origin is properly segregated.

Market: xxx City: Quezon City District V

Date: 22 May 2017

Time of inspection: 7:40 am

|  | **1** | **2** | **3** | **4** | **5** |
| --- | --- | --- | --- | --- | --- |
| **A. Lighting** |  |  | **X** |  |  |
| **B. Ventilation** |  |  | **X** |  |  |
| **C. Floorings** |  |  |  |  |  |
| Finished? |  | **X** |  |  |  |
| Moisture (Dry 🡪 Wet) |  |  |  |  | **X** |
| Cleanliness |  |  |  |  | **X** |
| **D. Display Tables** |  |  |  |  |  |
| Finished? |  | **X** |  |  |  |
| Smooth surface |  |  |  | **X** |  |
| Drains? |  |  |  | **X** |  |
| Elevation |  | **X** |  |  |  |
| Cleanliness |  |  |  | **X** |  |
| Moisture (*ice/water/blood*) |  |  |  |  | **X** |
| **E. Vendors/Food handlers** |  |  |  |  |  |
| Proper attire |  |  | **X** |  |  |
| Transact while selling |  |  | **X** |  |  |
| **F. Equipment (*cleanliness*)** |  |  |  |  |  |
| Knife |  |  | **X** |  |  |
| Grinder |  |  | **X** |  |  |
| Chopping board |  |  | **X** |  |  |
| **G. Zoning for whole market** |  |  |  |  |  |
| Meat and meat food  Products |  |  |  |  | **X** |
| Chicken (isolated) |  |  |  |  | **X** |
| Pork |  |  |  |  | **X** |
| Beef |  |  |  |  | **X** |
| **H. Pest** |  |  |  |  |  |
| Flies (absence 🡪 presence) |  |  | **X** |  |  |
| Cockroaches (absence 🡪  presence) |  |  |  |  |  |
| Rats |  |  |  |  |  |
| **I. Cleanliness** |  |  |  |  |  |
| Waste segregation |  |  |  | **X** |  |
| Trash can |  |  |  | **X** |  |
| Trash |  |  |  | **X** |  |
| Odor |  |  |  | **X** |  |
| **J. Cooling system** |  |  |  |  |  |
| Refrigerator/Chiller |  |  |  |  | **X** |
| Ice |  |  |  |  | **X** |
| **M. Sewage (presence)** |  |  |  |  |  |
| Clean |  |  |  |  | **X** |
| **N. Meat** |  |  |  |  |  |
| Handling |  |  | **X** |  |  |
| Freshness |  | **X** |  |  |  |
| **O. Meat stalls** |  |  |  |  |  |
| Separate from fish stalls |  |  |  |  | **X** |
| Evisceration, skinning | **X** |  |  |  |  |
| Chopping blocks, easily  cleaned, free of cracks and  crevices |  |  | **X** |  |  |
| Hang in hanging rails, not lying  on the counter |  |  | **X** |  |  |
| Clean |  |  |  |  | **X** |
| Free for vermin |  |  |  |  |  |
| Processing of meat (tocino,  longganisa) | Processing of longganisa was observed. | | | | |

1 – Very good 4 - Bad

2 – Good 5- Worst

3 – Neutral

Remarks:

- No proper segregation of products (dry, fresh produce, fish, and meat)
- Display area and floors are wet
- Some food are hanging, while others are lying on container (or on pans).

Markets: xxx City: Manila

Date: 2015

Time of inspection:-

|  | **1** | **2** | **3** | **4** | **5** |
| --- | --- | --- | --- | --- | --- |
| **A. Lighting** |  |  |  | **X** |  |
| **B. Ventilation** |  |  |  | **X** |  |
| **C. Floorings** |  |  |  |  |  |
| Finished? |  |  |  | **X** |  |
| Moisture (Dry 🡪 Wet) |  |  |  | **X** |  |
| Cleanliness |  |  |  | **X** |  |
| **D. Display Tables** |  |  |  |  |  |
| Finished? |  | **X** |  |  |  |
| Smooth surface |  |  | **X** |  |  |
| Drains? |  |  |  | **X** |  |
| Elevation |  | **X** |  |  |  |
| Cleanliness |  |  |  | **X** |  |
| Moisture (*ice/water/blood*) |  |  |  | **X** |  |
| **E. Vendors/Food handlers** |  |  |  |  |  |
| Proper attire |  |  | **X** |  |  |
| Transact while selling |  |  | **X** |  |  |
| **F. Equipment (*cleanliness*)** |  |  |  |  |  |
| Knife |  |  | **X** |  |  |
| Grinder |  |  | X |  |  |
| Chopping board |  |  | X |  |  |
| **G. Zoning for whole market** |  |  |  |  |  |
| Meat and meat food  Products |  |  | **X** |  |  |
| Chicken (isolated) |  |  | **X** |  |  |
| Pork |  |  | **X** |  |  |
| Beef |  |  | **X** |  |  |
| **H. Pest** |  |  |  |  |  |
| Flies (absence 🡪 presence) |  |  |  | **X** |  |
| Cockroaches (absence 🡪  presence) |  |  |  | **X** |  |
| Rats |  |  |  |  |  |
| **I. Cleanliness** |  |  |  |  |  |
| Waste segregation |  |  |  | **X** |  |
| Trash can |  |  |  | **X** |  |
| Trash |  |  |  | **X** |  |
| Odor |  |  |  | **X** |  |
| **J. Cooling system** |  |  |  |  |  |
| Refrigerator/Chiller |  |  |  |  | **X** |
| Ice |  |  |  |  | **X** |
| **M. Sewage (presence)** |  |  |  |  |  |
| Clean |  |  |  | **X** |  |
| **N. Meat** |  |  |  |  |  |
| Handling |  |  | **X** |  |  |
| Freshness |  | **X** |  |  |  |
| **O. Meat stalls** |  |  |  |  |  |
| Separate from fish stalls |  |  |  | **X** |  |
| Evisceration, skinning |  | **X** |  |  |  |
| Chopping blocks, easily  cleaned, free of cracks and  crevices |  |  | **X** |  |  |
| Hang in hanging rails, not lying  on the counter |  |  | **X** |  |  |
| Clean |  |  |  | **X** |  |
| Free for vermin |  |  |  |  |  |
| Processing of meat (tocino,  longganisa) |  | | | | |

1 – Very good 4 - Bad

2 – Good 5- Worst

3 – Neutral

Remarks:

- Quinta market is newly renovated, hence, present ocular inspection data were not useful for analysis.
- Data shown above were based from 2015 sampling.
